# Therapeutic Potential of EGCG, a Green Tea Polyphenol, for Treatment of Coronavirus Diseases

**DOI:** 10.3390/life11030197

**Published:** 2021-03-04

**Authors:** Junsoo Park, Rackhyun Park, Minsu Jang, Yea-In Park

**Affiliations:** Division of Biological Science and Technology, Yonsei University, Wonju 26493, Korea; rockhyun@yonsei.ac.kr (R.P.); minsujang@yonsei.ac.kr (M.J.); pyi012324@yonsei.ac.kr (Y.-I.P.)

**Keywords:** coronavirus, COVID-19, SARS-CoV-2, EGCG, green tea

## Abstract

Epigallocatechin gallate (EGCG) is a major catechin found in green tea, and there is mounting evidence that EGCG is potentially useful for the treatment of coronavirus diseases, including coronavirus disease 2019 (COVID-19). Coronaviruses encode polyproteins that are cleaved by 3CL protease (the main protease) for maturation. Therefore, 3CL protease is regarded as the main target of antivirals against coronaviruses. EGCG is a major constituent of brewed green tea, and several studies have reported that EGCG inhibits the enzymatic activity of the coronavirus 3CL protease. Moreover, EGCG has been reported to regulate other potential targets, such as RNA-dependent RNA polymerase and the viral spike protein. Finally, recent studies have demonstrated that EGCG treatment interferes with the replication of coronavirus. In addition, the bioavailability of EGCG and future research prospects are discussed.

## 1. Introduction

The coronavirus disease 2019 (COVID-19) pandemic has impacted all aspects of society, leading to extensive investigations into remedies for severe acute respiratory syndrome coronavirus 2 (SARS-CoV-2), the causative virus of COVID-19. As of 2021, vaccines for SARS-CoV-2 are available, but effective antiviral medicines for COVID-19 are not yet available. Since the vaccination speed is limited, additional years will be required to achieve complete herd immunity. Moreover, it is expected that novel coronavirus diseases will emerge in the future. Therefore, numerous antiviral medicines should be developed to treat or alleviate coronavirus diseases.

There are four coronavirus subfamilies: alpha, beta, gamma and delta; and seven coronaviruses are known to infect human [1]. SARS-CoV-2, Middle East respiratory syndrome (MERS) and SARS-CoV belong to beta coronaviruses and can cause severe disease [2]. There are four additional human coronaviruses and these human coronaviruses are associated with mild symptoms. Human coronavirus OC43 (HCoV-OC43) and HCoV-HKU1 belong to beta coronaviruses and HCoV-229E and HCoV-NL63 belong to alpha coronaviruses [3].

**Figure 1:**
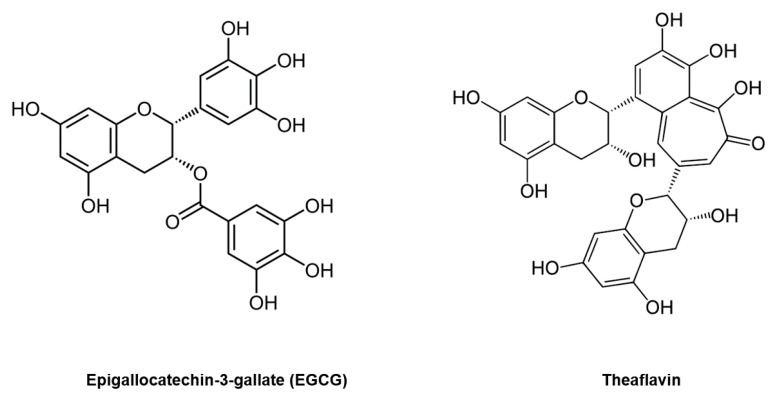
Molecular structures of epigallocatechin-3-gallate (EGCG) and theaflavin.

Green tea has been a popular beverage for millennia, and many reports have shown that drinking green tea has various health benefits, such as cancer prevention and treatment of infectious diseases [4,5]. Epigallocatechin-3-gallate (EGCG) is the major catechin in green tea, accounting for 50–80% of the catechins in a brewed cup of green tea, and one cup of green tea contains approximately 100–300 mg of EGCG [6,7,8] (Figure 1). In black tea, theaflavin is the major constituent, and theaflavin has also shown several beneficial effects [9] (Figure 1). EGCG absorption is relatively high, and its maximum plasma concentration exceeds 1 μg/mL [4]. However, the absorption of theaflavin is poor, and its bioavailability is much lower than that of EGCG [10,11]. Therefore, in this review, we have focused on the effects of EGCG against coronavirus diseases.

Recently, one study examined the relationship between green tea consumption and overall COVID-19 risk, including morbidity and mortality [12]. Although there were huge socioeconomic differences among study participants, increased green tea consumption resulted in significantly low COVID-19 morbidity and mortality [12]. Because this preliminary epidemiological study used publicly available statistical data, we also obtained comparable results by analyzing similar datasets. These statistics suggest that green tea or green tea components have the potential to treat or prevent COVID-19 and potentially other coronavirus diseases. In this review, we discuss the potential role of EGCG, a major green tea component, in the prevention and treatment of coronavirus diseases. We performed a systematic review of the literature using PubMed and Google scholar till February 2021 for published papers and preprints on EGCG and coronavirus.

## 2. 3-Chymotrypsin-Like (3CL) Protease is the Major Therapeutic Target for Antivirals to Treat Coronavirus Disease

Many antiviral medicines have been developed to treat viral diseases, and virus-specific enzymes are major targets for drug development. For example, human immunodeficiency virus (HIV) encodes reverse transcriptase and protease, and inhibitors of these enzymes are well-known antiviral drugs for acquired immune deficiency syndrome (AIDS) [13]. Likewise, coronavirus encodes an RNA-dependent RNA polymerase (RdRp) and proteases [14]. Several RdRp-targeting drugs, such as ribavirin and remdesivir, have been developed and tested to treat SARS, MERS, and SARS-CoV-2 [15]. However, limited therapeutic effects have been reported in clinical trials [16,17].

**Figure 2:**
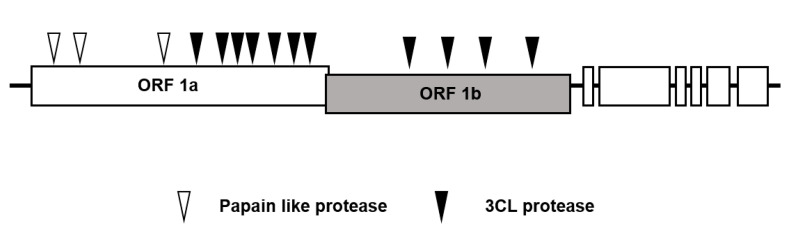
Cleavage of coronavirus polyproteins by 3-chymotrypsin-like-protease (3CL protease).

Coronavirus-specific proteases are candidate targets for viral drug development [18]. Coronavirus encodes polyproteins, and viral proteases cleave these polyproteins into individual functional proteins [19]. The 3-chymotrypsin-like-protease (3CL protease or main protease) and papain-like protease are responsible for the cleavage of polyproteins by cleaving 11 sites and 3 sites, respectively (Figure 2) [20]. Because 3CL protease cleaves more polyprotein sites, 3CL protease is the preferred candidate for antiviral medicine development [18,21,22]. In early 2020, the 3D structure of SARS-CoV-2 3CL protease was determined, and peptidomimetic inhibitors showed inhibitory effects on 3CL protease enzyme activity as well as on SARS-CoV-2 replication [23]. In addition, lopinavir and ritonavir, which were reported to repress 3CL protease, were investigated as potential medicines for the treatment of COVID-19 [24,25,26,27]. However, lopinavir-ritonavir treatment did not provide a significant benefit to patients with COVID-19 in clinical trials [28,29]. Later, an in vitro enzymatic assay demonstrated that lopinavir-ritonavir did not efficiently inhibit SARS-CoV-2 3CL protease [30]. These results suggest that 3CL protease is a validated target for coronavirus antiviral medicines.

## 3. Epigallocatechin-3-Gallate (EGCG) Inhibits Coronavirus 3CL Protease

EGCG is a major constituent of green tea, and EGCG treatment has been reported to inhibit various viruses [31]. Previous studies have reported that EGCG inhibits the hepatitis C virus and human immunodeficiency virus from entering host cells by interfering with virus–cell receptor interactions [32,33]. In addition, EGCC treatment also inhibits infection by the influenza, Ebola, and dengue viruses [34,35,36].

Because 3CL protease is essential for coronavirus maturation, potential inhibitors of 3CL protease were sought to develop antivirals for coronavirus. Initially, SARS 3CL protease was used to screen the inhibitors using a 720-compound library, and tea components, including tannic acid and 3-isotheaflavin-3-gallate (TF2B), showed inhibitory effects against SARS 3CL protease [37]. Several tea extracts and tea components were examined for their inhibitory effects on 3CL protease, and EGCG showed a relatively weak inhibitory effect [37]. Later, yeast expressing the SARS 3CL protease was used to confirm the inhibitory effect of EGCG against 3CL protease [38]. Because SARS is not widely prevalent like SARS-CoV-2, there have been few studies on EGCG and the SARS coronavirus. In 2020, COVID-19 became a global pandemic, and the causal virus, SARS-CoV-2, has attracted the attention of researchers. We cloned SARS-CoV-2 3CL protease cDNA by chemical synthesis and obtained SARS-CoV-2 3CL protease protein using a bacterial expression system [39]. We also screened potential inhibitors and found that green tea showed a strong inhibitory effect against SARS-CoV-2 3CL protease. Finally, we demonstrated that the tea polyphenols, EGCG and theaflavin, exert inhibitory effects against SARS-CoV-2 3CL protease [39]. Similar results were obtained from other research groups, confirming that EGCG inhibits the SARS-CoV-2 3CL protease [40,41,42].

Notably, the SARS-CoV-2 3CL protease appeared to be more sensitive to EGCG than the SARS 3CL protease (Table 1). The half-maximal inhibitory concentration (IC_50_) of EGCG for SARS 3CL protease ranged from 25 μM to greater than 100 μM, and the IC_50_ for SARS-CoV-2 3CL protease ranged from 0.847 μM to 16.5 μM, according to our results (Table 1). In addition, we examined the inhibitory effects of EGCG against HCoV-OC43 and HCoV-229E, which belong to the human coronaviruses; the ECGC IC_50_ values for HCoV-OC43 and HCoV-229E were greater than the IC_50_ for SARS-CoV-2 [43]. These results suggest that EGCG treatment is potentially more effective against SARS-CoV-2 than SARS and other coronaviruses. 

In addition to the 3CL protease enzymatic assay, structural analysis supports EGCG being a potential SARS-CoV-2 3CL protease inhibitor. In silico molecular docking studies revealed that EGCG interacts with the catalytic residues of 3CL protease, and the 3CL protease-EGCG interaction is highly stable, indicating that EGCG shows drug-like characteristics toward 3CL protease [44,45,46]. Moreover, several in silico structural models confirmed that EGCG specifically binds to the 3CL protease [44,46]. Taken together, EGCG specifically interacts with the coronavirus 3CL protease and inhibits 3CL protease activity in vitro.

## 4. Possible Regulation of other Targets besides 3CL Protease by EGCG

While the coronavirus 3CL protease has been intensively studied as a target of EGCG, additional coronavirus targets have been proposed. Since coronavirus is an RNA virus, RNA-dependent RNA polymerase (RdRp) is a popular target of antiviral medicines [47]. Polyphenols, including EGCG, have shown inhibitory effects on the RdRp of the influenza A virus [48]. In silico structural analysis has suggested that EGCG interacts with SARS-CoV-2 RdRp and forms a stable complex, indicating that EGCG potentially interferes with SARS-CoV-2 RdRp function [49].

The SARS-CoV-2 spike glycoprotein is known to interact with the host cell angiotensin-converting enzyme 2 (ACE2) protein to initiate viral infection. Therefore, SARS-CoV-2 infection can be prevented by blocking the interaction between the viral spike protein and ACE2. Several structural studies have suggested that EGCG interacts with the SARS-CoV-2 spike proteins [46,50]. Moreover, pretreating vesicular stomatitis virus expressing coronavirus spike protein with green tea extract or EGCG inhibits viral entry into host cells [51]. In addition, nuclear factor erythroid-derived 2-related factor 2 (NRF2) is known to reduce the expression of ACE2, a receptor for SARS-CoV-2 infection in lung epithelial cells, and decreased levels of NRF2 may contribute to efficient SARS-CoV-2 infection [52,53]. Therefore, a hypothesis paper suggested that the activation of NRF2 protein by treatment with EGCG, a known NRF2 activator, can downregulate SARS-CoV-2 infection [52]. These studies collectively suggest that EGCG potentially interferes with coronavirus entry into the host cell by modulating the viral spike protein and ACE2 interaction. 

Finally, immune modulation can be a target of EGCG in the treatment of coronavirus diseases. Patients with COVID-19 can develop acute pneumonia, which can lead to the onset of a cytokine storm resulting from the upregulation of pro-inflammatory cytokines [54]. Since EGCG is reported to have anti-inflammatory activity, EGCG treatment could counteract the massive production of cytokines by regulating STAT1/3 and NF-κB signaling [55]. EGCG treatment can also upregulate interferon λ1 signaling, which is responsible for antiviral functions [56]. These findings suggest that EGCG treatment can relieve coronavirus symptoms by modulating the immune system.

## 5. EGCG Inhibits Coronavirus Replication

Since EGCG treatment showed inhibitory effects on 3CL protease and ACE2 binding in vitro, EGCG was expected to inhibit coronavirus replication. However, the handling of SARS or SARS-CoV-2 requires a BSL-3/BSL-4 lab facility, and the use of these facilities is limited. For this reason, there are limited experimental infection data for SARS or SARS-CoV-2 to support the role of EGCG in coronavirus replication (Table 2). 

Bovine coronavirus is a causative virus for diarrhea in cattle, and pretreatment with EGCG significantly decreases the propagation of bovine coronavirus in host cells [57]. Incubation of EGCG (0.5–10 μg/mL) with bovine coronavirus efficiently inhibits coronavirus propagation [57]. Recently, we demonstrated that EGCG treatment significantly blocks the replication of HCoV-229E and HCoV-OC43 in a dose-dependent manner [43]. Notably, EGCG treatment slightly increased the coronavirus RNA copy number in the infected cells; we speculate that EGCG interferes with the release of the viral RNA genome [43]. Finally, SARS-CoV-2 was used to examine the inhibitory effect of EGCG on coronavirus replication, and pretreatment of EGCG with SARS-CoV-2 significantly blocked coronavirus replication [58]. In addition, vesicular stomatitis virus pseudotyped with the SARS-CoV-2 spike protein was used to examine the inhibitory effect of EGCG on the interaction between the SARS-CoV-2 spike protein and ACE2; EGCG treatment efficiently blocked infection by the vesicular stomatitis virus pseudotyped with the coronavirus spike protein [51]. These results indicate that EGCG can block coronavirus infection and coronavirus replication. Notably, the IC_50_ of EGCG for coronavirus replication was relatively lower than that for 3CL protease inhibition. Since the coronavirus polyprotein contains multiple cleavage sites for 3CL protease, partial inhibition of polyprotein cleavage by EGCG may contribute to the inhibition of coronavirus replication [43]. The inhibition of other targets, such as RdRp or ACE2, may also contribute to the inhibition of coronavirus by EGCG. Collectively, these results indicate that EGCG has the potential to block coronavirus replication. 

## 6. In Vivo Distribution of EGCG

Although EGCG efficiently inhibits coronavirus 3CL protease and replication in vitro, it is unknown whether EGCG can reach an effective concentration in vivo. For this reason, we conducted a literature review of the known in vivo distributions of EGCG. Although the respiratory tract is the primary site for SARS-CoV-2 infection, recent data indicate that SARS-CoV-2 also infects the gastrointestinal tract [59]. Moreover, recent reports have shown that gastrointestinal illness is associated with enteric SARS-CoV-2 infections [60,61]. SARS-CoV-2 is frequently found in the stool of patients with COVID-19, suggesting that it can be transmitted via a fecal-oral route [61,62]. Although respiratory transmission is the major infection route for SARS-CoV-2, fecal-oral transmission may be an alternative route by which coronavirus can spread [63,64].

**Figure 3:**
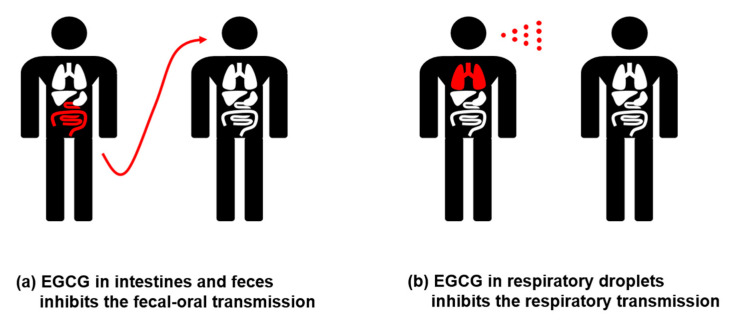
Possible inhibitory mechanisms of EGCG in the transmission of coronavirus.

Since SARS-CoV-2 is detected in both the respiratory and gastrointestinal tracts, the distribution of EGCG in the lung, intestine, and colon is summarized in Table 3. EGCG can be administered intravenously via an injection or orally. Although intravenous injection of EGCG can increase the concentration of EGCG in the lungs, oral administration of EGCG results in a relatively low concentration of EGCG in the lungs [65]. The maximum observed concentration of EGCG in the lungs was less than 2 μg/g [65]. However, the distribution of EGCG in the small intestine and colon was higher than the IC_50_ for 3CL protease inhibition [65,66]. In particular, oral administration of EGCG resulted in higher levels of EGCG in the intestine and colon [65,66]. Since a small proportion of EGCG is absorbed in the intestine, most of the EGCG localizes in the feces, and the concentration of EGCG in feces is even higher than that in tissues. Therefore, we speculate that EGCG can inhibit coronavirus replication in enteric sites and patient feces, and fecal-oral transmission will be prevented by excessive concentrations of EGCG at these sites (Figure 3). In addition, the accumulation of EGCG through its repeated consumption may enhance the distribution of EGCG in other tissues.

Saliva is important for the respiratory transmission of coronavirus because coronavirus is transmitted primarily through respiratory droplets, which contain saliva [69]. When one drinks green tea, the amount of EGCG in saliva is significantly increased, and the level of EGCG can reach the effective concentrations required to inhibit 3CL protease and coronavirus replication [68]. Recent reports indicate that drinking tea or gargling with tea rapidly inactivate coronavirus infectivity in saliva, thereby making it possible to attenuate the spread of SARS-CoV-2 [70]. These results suggest that adding EGCG to saliva may offer an additional means of preventing coronavirus infections and transmission (Figure 3).

## 7. Conclusion and Perspective

Here, we have reviewed recent research progress on the therapeutic potential of EGCG for treating coronavirus diseases. EGCG is an abundant tea polyphenol in brewed green tea, and many reports provide evidence that EGCG can efficiently block 3CL protease, an essential enzyme for coronavirus replication [6,39]. Moreover, additional coronavirus targets of EGCG such as RdRp and spike protein have been proposed [49,51]. Finally, EGCG treatment interferes with the replication of coronaviruses in cell culture systems [43]. When the in vivo distribution of EGCG was investigated, the concentration of EGCG in the intestine and colon was higher than most of the concentrations (i.e., the IC_50_ values) required to effectively inhibit 3CL protease [65,66]. In addition, coronavirus polyproteins contain 11 cleavage sites, and a lower concentration can be effective in treating coronavirus diseases [43]. Likewise, a preliminary statistical study suggested that green tea consumption could reduce the overall risk of coronavirus [12]. These results collectively support the idea that EGCG is potentially effective for the treatment of coronavirus diseases.

Because most of the EGCG data were obtained from in vitro studies, animal experiments or clinical tests are required to confirm the effects of EGCG on coronavirus diseases. Since green tea comprises EGCG as the main constituent, its extract can be used in in vivo experiments. As the safety of green tea has long been verified, an adequate amount of green tea can be directly used in in vivo experiments without toxicity concerns. These experiments will determine whether EGCG or green tea is useful for the treatment of coronavirus diseases. In addition, an epidemiological study would be useful for examining the effects of EGCG or green tea on coronavirus diseases. Although preliminary statistical results are available, the results of epidemiological studies, such as the correlation between personal green tea consumption and the risk of developing coronavirus disease, can be evaluated to determine the effects of green tea on coronavirus [12]. We expect that more researchers will become interested in EGCG and perform extensive research to confirm the therapeutic effects of EGCG on coronavirus diseases. We also caution that EGCG should not be used as a treatment for COVID-19 until further clinical studies occur.

## Figures and Tables

**Table 1:** IC_50_ of EGCG for coronavirus 3CL protease.

Virus	IC_50_	References
SARS-CoV-2	7.58 μg/mL (16.5 μM)	[39]
SARS-CoV-2	4.24 μM	[40]
SARS-CoV-2	7.51 μM	[41]
SARS-CoV-2	0.847 μM	[42]
SARS-CoV	24.98 μM	[40]
SARS-CoV	>100 μM	[37]
SARS-CoV	73 μM	[38]
HCoV-OC43	14.6 μg/mL (31.8 μM)	[43]
HCoV-229E	11.7 μg/mL (25.5 μM)	[43]

**Table 2:** Summary of coronavirus replication inhibition by EGCG.

Virus	Description	References
Bovine coronavirus	Treatment of bovine coronavirus with EGCG (5 μg/mL) decreases plaque numbers by up to 80%.	[57]
HCoV-OC43	EGCG treatment decreases coronavirus protein in infected cell media, with an IC_50_ of approximately 1–5 μg/mL.	[43]
HCoV-229E	EGCG treatment decreases coronavirus RNA in infected cell media, with an IC_50_ of 6.92–8.73 μg/mL.	[43]
Vesicular stomatitis virus pseudotyped with SARS-CoV-2 spike protein	Treatment of EGCG (100 μg/mL) inhibits viral infection by up to 90%.	[51]
SARS-CoV-2	Treatment of SARS-CoV-2 with EGCG (100 μM) significantly decreases viral RNA in infected cell media.	[58]

**Table 3:** Amount of EGCG in coronavirus-related tissues.

Animal	Tissue	Administration	Maximum Concentration	References
Rat	Lung	oral	<2 μg/g *	[67]
Rat	Lung	intravenous	2.66 nmol/g (1.22 μg/g)	[65]
Rat	Lung	oral	0.01 nmol/g (0.0045 μg/g)	[65]
Rat	Small intestinal mucosa	oral	565 nmol/g (259 μg/g)	[66]
Rat	Small Intestine	oral	45.2 nmol/g (20.7 μg/g)	[65]
Rat	Intestine	oral	10–25 μg/g *	[67]
Rat	Small Intestine	intravenous	2.4 nmol/g (1.1 μg/g)	[65]
Rat	Colon mucosa	oral	68.6 nmol/g (31.4 μg/g)	[66]
Rat	Colon	intravenous	1.2 nmol/g (0.55 μg/g)	[65]
Rat	Colon	oral	7.9 nmol/g (3.6 μg/g)	[65]
Human	Saliva	oral	4.8–22 μg/mL	[68]

* Values were estimated from the original graph.

## Data Availability

Not applicable.
